# Use of Mobile Phones as Intelligent Sensors for Sound Input Analysis and Sleep State Detection

**DOI:** 10.3390/s110606037

**Published:** 2011-06-03

**Authors:** Ondrej Krejcar, Jakub Jirka, Dalibor Janckulik

**Affiliations:** 1 Department of Information Technologies, Faculty of Informatics and Management, University of Hradec Kralove, Rokitanskeho 62, Hradec Kralove 50003, Czech Republic; 2 Department of Measurement and Control, Faculty of Electrical Engineering and Computer Science, VSB Technical University of Ostrava, 17. Listopadu 15, Ostrava Poruba 70833, Czech Republic; E-Mails: Jakub.Jirka1@vsb.cz (J.J.); Dalibor.Janckulik@vsb.cz (D.J.)

**Keywords:** sleep stages detection, hypnogram, Windows Mobile, FFT analysis

## Abstract

Sleep is not just a passive process, but rather a highly dynamic process that is terminated by waking up. Throughout the night a specific number of sleep stages that are repeatedly changing in various periods of time take place. These specific time intervals and specific sleep stages are very important for the wake up event. It is far more difficult to wake up during the deep NREM (2–4) stage of sleep because the rest of the body is still sleeping. On the other hand if we wake up during the mild (REM, NREM1) sleep stage it is a much more pleasant experience for us and for our bodies. This problem led the authors to undertake this study and develop a Windows Mobile-based device application called wakeNsmile. The wakeNsmile application records and monitors the sleep stages for specific amounts of time before a desired alarm time set by users. It uses a built-in microphone and determines the optimal time to wake the user up. Hence, if the user sets an alarm in wakeNsmile to 7:00 and wakeNsmile detects that a more appropriate time to wake up (REM stage) is at 6:50, the alarm will start at 6:50. The current availability and low price of mobile devices is yet another reason to use and develop such an application that will hopefully help someone to wakeNsmile in the morning. So far, the wakeNsmile application has been tested on four individuals introduced in the final section.

## 1. Introduction

Sleep is a complex process regulated by our brain and as such is driven by a 24 h biological rhythm. As we age, our sleeping habits change rapidly. A newborn baby sleeps in short periods for 18 h a day. According to studies, 3 h of sleep is enough for some people, but others need eight or even 10 h of sleep to feel rested. Recent research [1] shows that differences in the biological rhythm of people can explain why some teenagers have such difficulty in waking up. It seems that during puberty this rhythm shifts and adults tend to go to bed much later and to wake up much later as well. This sleep shift is common and during adolescence it disappears [2]. Our biological clocks are controlled by chemical substances that are mostly known to us. One of them is hormone called melatonin, which is suspected to make us feel sleepy. This substance is produced in our brain and some scientists believe that it is also causes our metabolism to slow down before falling asleep. Melatonin secretion leads to a reduction of body temperature, a limitation of blood flow towards brain and a slackness of muscles [3].

### 1.1. Sleep Stages

Approximately 2 h after we fall asleep our eyes start to move back and forth irregularly. Based on this fact scientists have divided sleep stages into two main stages, namely REM sleep with (Rapid Eye Movement) and NREM sleep stage (Non Rapid Eye Movement). NREM sleep is divided into another four sub-stages in which a sleep gets gradually deeper and deeper [4]. During a healthy sleep, REM and NREM stages interchange several times. Most of the dreams happen during the REM stage. Body muscles are completely relaxed and as a result, waking up at this stage is refreshing. During the deep sleep stages (NREM 3 and 4), blood pressure is decreasing, which lowers the risk of cardiovascular disease [3] and, in adolescence, a growth hormone is produced at maximum levels [5]. To summarize, the sleep stages are as follows:

- Wake (Awake)
- REM—we dream in this stage
- NREM1—falling asleep
- NREM2—light sleep
- NREM3—deep sleep
- NREM4—deepest sleep

Table 1 describes and outlines the processes in the human body during the individual sleep stages. It shows the relationships between biological manifestations and the different sleep stages [3]. This table and information therein are taken into account in the wakeNsmile detection algorithm.

### 1.2. Hypnogram

**Figure 1.**
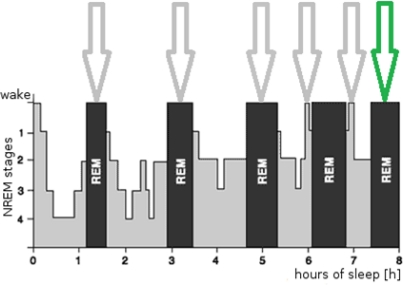
Hypnogram with optimal wake up periods.

A hypnogram is a graph that visualizes sleep profile in time (as a series of individual stages). According to information outlined above, the optimal moments to wake up, corresponding to periods of mild sleep, are denoted in Figure 1.

The most optimal period of time is of course after 8 h of sleep, marked with a green arrow in Figure 1. The sleep is recorded and analyzed in the time slot marked by the green arrow and if mild-sleep event is identified based on factors described in Section 1, the user is awakened. The next section describes the analysis algorithm that is used to detect mild sleep stages using recorded sound as a data source that is processed.

## 2. Related Work

The final goal of this project was to develop a Windows Mobile application which will implement algorithms to detect sleep stages. Currently, some similar applications are available for download that run on various mobile device platforms from Nokia, Apple iPhone, Windows Mobile, *etc*. However, developers typically do not provide information on the algorithms involved, how are they implemented, how many sleep stages they detect accurately and how many users does it wake up at the right time.

One such application [6] is available for Nokia and iPhone mobile platforms. HappyWakeUp *“does not try to wake you up when you are in deep and calm sleep or if you are in REM sleep (sleep phase with dreaming). These are the moments when it is most difficult to wake up”* [6]. The HappyWakeUp application is based on actively monitoring the sleep of users via the microphone of their mobile phone. It attempts to detect users’ movements in bed. This is the reason why the user’s mobile device (mobile phone) must be placed near the user. The application then conducts a statistical analysis of the quality and cycles of the user’s sleep. As the authors [of this application] describe it, the application is developed to detect only statistically significant movements such as arousals, because during these moments users are actually awake or almost awake [6]. However, the authors have not published any other details on the algorithms or techniques, so it is impossible to evaluate their software according to these principles.

It is also possible to find other applications and projects similar to HappyWakeUp. For example, Macjek Drejak Labs developed a mobile application called Sleep Cycle. This application *“…has become a huge success with a #1 paid app position in many countries, including Germany, Japan and Russia”* [7]. Unlike HappyWakeUp, Sleep Cycle monitors a user’s moves by using the embedded accelerometers which are fitted in some modern mobile devices (e.g., iPhones or some HTC phones). During different sleep stages the user makes different moves in bed and the movement patterns can be determined by Sleep Cycle. The other stages of signal processing remain unchanged. The next section deals with algorithms and their implementation to real applications to detect sleep stages by using microphone input. The use of accelerometers will be investigated in the near future.

## 3. Sleep Stage Analysis Algorithm

The sleep stage analysis algorithm is based on knowledge about specific sleep stages summarized in Table 1 and on empirical testing on several selected subjects results.

A detection algorithm is based on an assumption that if a patient is in the REM phase and his sleep is not disturbed or pathological in any way, his muscles start to spasm, which leads to movements that can be recorded and detected by the device microphone. If the patient is in NREM stage 2 or any higher sleep stage, all his muscles are relaxed and thus no body movement occurs [8].

By default the recording itself is triggered 30 min before the wake up time as set by the user before going to sleep. If a REM stage movement is detected within this interval, the user is awakened at the time when this movement occurs.

The time interval of 30 min was estimated on the basis of the facts mentioned in studies [3,5] and on empirical testing. This time window represents the REM stage of sleep, when we are closest to being awake. If no movement sound is detected during this 30 min window, the user is awakened at the set time. According to [3], at least one REM phase occurs during this 30 min window.

The size of the wake up window can be adjusted by the programmer who can change a WAKE_WINDOW_LENGTH constant, found in the Analyzer class. The user cannot change the size of this window as it is the key factor for the analysis algorithm.

There are areas where the detection algorithm does not work or where some precautions need to be taken to make the algorithm usable. This method is not applicable for people with sleeping disorders like insomnia, hypopnea syndrome, parasomnia, periodic limb movement disorder (PLMD), *etc*., whose sleeping manifestations are pathological and unsuitable for the detection algorithm. This method is also not applicable in noisy areas, which in any case are also unhealthy for sleeping.

Another problem is when there are pathological sound manifestations of sleep like snoring or cough present. These kinds of manifestations can be detected and filtered out. Snoring itself is a physiological periodic process that can be detected and distinguished from the REM phase muscle spasm movement anomaly sounds which are the key for the detection algorithm. Snoring is an unwanted event that occurs during NREM sleep stages [9].

From the signal analysis capability point of view, sound signals that correlate with sleep stages and their manifestations can be divided into three groups.

- Pathological sleep sound manifestation signals that are not suitable for further analysis (insomnia, PLMD, …) and noisy areas.
- Pathological sleep sound manifestation signals that are acceptable for further processing (snoring, coughing).
- Physiological healthy sleeping with correct REM and NREM sleep stages sound manifestations suitable for further processing.

The last two groups are processed in the detection algorithm and described in the following sections.

### 3.1. Filterable Pathological Manifestation Sounds—Pathologicals Filter

**Figure 2.**
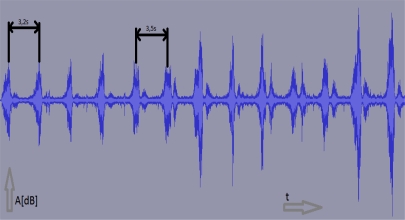
Example of snoring sound obtained during sleep.

From the signal analysis point of view, snoring is a periodical signal with similar amplitudes. Figure 2 shows an example of snoring during the sleep of an individual with higher respiratory resistance. As seen in the figure, the snoring sound is periodic with very small deviations and clearly distinguishable from other signals. Snoring is detected by the algorithm as a periodical event with amplitudes with small deviations.

**Figure 3.**
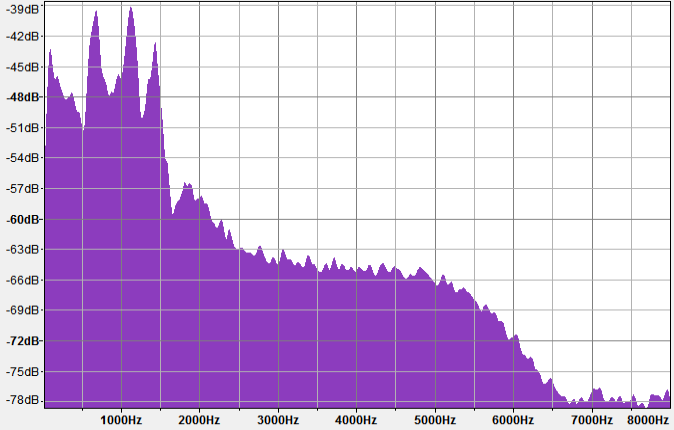
FFT of signal from Figure 2.

Periodicity and amplitude similarity are not precise. Both have certain deviations which are taken into account. Time sample differences of snoring or other pathological events are always within 15% of a deviation (Figure 2 and Table 2) as tested on all subjects with pathological snoring sleep. The pathological filter algorithm operates in a time domain and not in a frequency domain due to the higher CPU time cost required for a FFT algorithm (Figure 3).

The snoring filter algorithm is based on the following facts:

- Time sample value T_X_ is taken from a signal peak.
- Amplitude value A_X_ is a peak value at a time sample stamp.
- If the last two time sample difference T_D_ is within 15% deviation of the total time difference calculated as a moving average of the last five time samples T_A_, then the time sample stamp is classified as snoring S and taken out from further processing.
- If the signal at a time sample stamp T_X_ is classified as snoring S, amplitude A_X_ is checked for the difference within 50% deviation of the last maximum signal A_M_.

$$\begin{array}{cc} T_{D}=T_{\left(n+1\right)}-T_{n},n\in Z & \text{Equation (1) Last two samples diff}.\\ T_{A}=\frac{\sum_{x=n-5}^{n}T_{D}{}_{{}_{X}}}{5},n\in Z & \text{Equation (2) Samples diff. MA}\\ S=\{|T_{D}-T_{A}|\;\;<\;\;0.15T_{A}\} & \text{Equation (3) Snoring class}.\text{set}\\ A_{C}=\{|A_{X}-A_{M_{{}_{n-1}}}|\;\;<\;\;0.5A_{M}{}_{{}_{n-1}}\} & \text{Equation (4) Amp.class}. \end{array}$$

### 3.2. Environmental Noise and Other Interferences

Every area where the patient is sleeping and where is he being recorded is typically full of noise and other interfering signals. These signals need to be filtered out in order to ensure the necessary signal quality for further REM/NREM stage signal analysis and detection. A FIR noise cancelation filter is used in this application. In this case the Math.NET Neodym [10] adaptive FIR filter library functions are used to filter out environmental noises. The LMS (Least Mean Squares) method is used to adapt filter coefficients recursively. This method is one of the most favored coefficient adaptation method for its simplicity and steep descent. Equations (1) [11] describing the adaptive general transversal filter are shown below. The filter equation is supplemented with an LMS algorithm update step Δ(n) describing the adaptation rate of a filter.

(1) $$\begin{array}{l} \text{y}(\text{n})=\text{d}(\text{n})-{\text{w}}^{\text{T}}(\text{n}-1)\text{u}(\text{n})\\ \text{w}(\text{n})=\text{w}(\text{n}-1)+\Delta (\text{n})\text{y}(\text{n})\\ \Delta (\text{n})=\mu \text{u}(\text{n}) \end{array}$$

**Figure 4.**
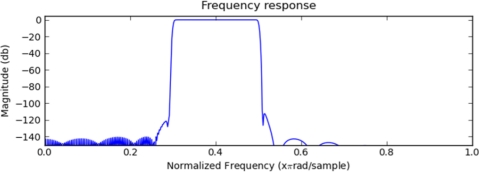
Used FIR filter window function.

The spectrum of frequencies below 10 Hz and above f = 1,500 Hz (Figure 3) are not desired as they do not contain any valid user movement information, only noise information and no higher frequencies have movement information and thus they are filtered out. A filter cutoff frequency value of 1,500 Hz has been estimated from many recorded samples of sleeping individuals and from all available testing devices mentioned in Table 2. Meth.Net algorithms are using windowed-sinc family Blackman-Harris window function described with Equation (2) and displayed in (Figure 4).

(2) h(n)=Bsinc(Bn)

### 3.3. Physiological Detectable Sounds

This section describes the algorithm used to detect the user REM stage movements during sleep. Data detected by this algorithm are already filtered and are classified to be processed. The algorithm itself is based on a time-series recorded data analysis. It uses real time analysis (time, amplitude) of sound waves that are abnormal and above an isoline. The isoline is computed by a linear moving average algorithm as in noisy areas and thanks to the different noise levels of the input devices the isoline might be above 0. The difference method between the real time data and the moving averaged data filters out both these problems. Waves that are likely to be more similar within biological manifestations of sleep (breath, snoring) are much more similar in means of period and amplitude and are filtered out by the pathological filter described in Section 3.1 (these waves as biological manifestations during sleep are taken as false deviations from the isoline as they most likely represent deeper NREM stages of sleep and thus are not valid to wake user up and are taken out of the final statistical analysis).

**Figure 5.**
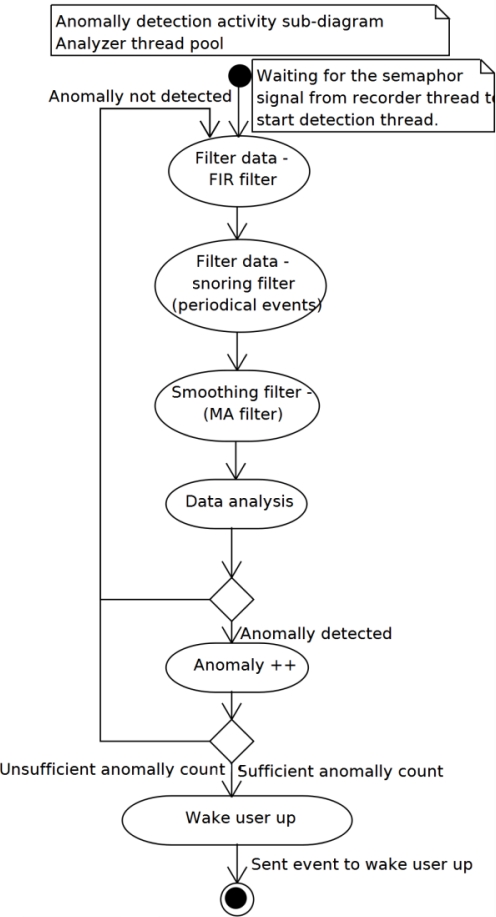
Analysis thread UML activity diagram.

On the other hand, if erratic waves, waves different in period and amplitude from the isoline are detected and registered at least DETECT_ANOMALIES_COUNT times, they are taken into the final statistical analysis as a positive sleep anomaly. These erratic waves in the final analysis most likely represent some user movement in a bed which is a manifestation of brain-motor interconnection that occur during the REM stage of the sleep and are valid and used to wake the user up. Figure 5 describes the whole detection algorithm by means of UML activity diagram.

## 4. WakeNsmile Application

**Figure 6.**
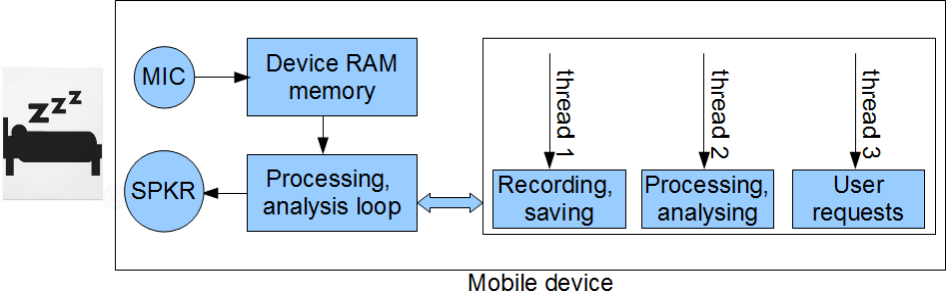
Block diagram of wakeNsmile application.

The WakeNsmile application (Figure 6) is written in C# programming language and uses. NET Compact Framework version 3.5, which is a special derivation of the .NET Framework for Mobile Devices [10,11]. The application was developed in Visual Studio 2008 Team Edition on Windows Mobile 6.5 emulator. A number of tests with several types of PDA devices have been undertaken (Table 3).

The minimum requirements for this application are mobile devices with Windows Mobile 6 and higher and .NET Compact Framework 3.5 or higher.

### 4.1. Design

**Figure 7.**
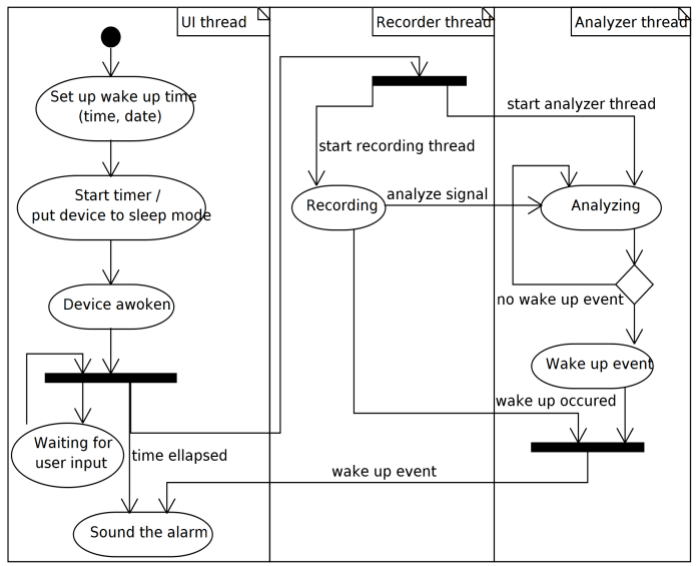
Application activity diagram.

The WakeNsmile application uses a user control called Alarm that has been created as a part of this project. The application uses the Math.NET [10] neodym library for FIR (Finite Impulse Response) filter design [12] and WaveIn and WaveOut libraries [13,14] for mobile device sound interface communication. The application structure is designed for real time recording and processing. The top level application structure is displayed in Figure 6. The top level application activity diagram displayed in Figure 7 shows how the threads from the application block diagram (Figure 6) are started and synchronized.

### 4.2. Implementation

The application uses a user control alarm created as a part of this project. This component is reusable and can be added to a newly created project and adjusted to the needs of a programmer. The user control employs a number of other classes and libraries suitable for signal recording and analysis:

- Recorder class
- Analyzer class
- Wave, WaveIn, WnsWaveIn, WaveOut class
- Player class

Recorder, Analyzer and Alarm (user control) classes are all running in their own threads so that the signal recording is not influenced by the signal analysis and the user is always able to interact with the program, even during the signal recording and analysis.

The Recorder class uses a WnsWaveIn class [15] and saves the sound recorded from the device microphone input. The class is designed using a singleton design pattern [11] because the device can only record from one device input at a time. An instance of a recorder class is running in its own thread, thus the graphical user interface Alarm (user control) is ready and available to receive commands at any time, including during the recording and analysis process, and an Analyzer class is able to analyze data samples from another sample recording without interrupting recording.

The .NET compact framework does not offer any classes or interfaces to communicate with the audio interface of the mobile device, consequently the WaveIn and WaveOut classes are used. These classes create an interface with the coredll.dll library which is an in/out device provider on the mobile device (an alternative to WindowsNT and higher kernel32.dll).

The Recorder class contains an important constant MAX_REC_TIME, which defines the time range of a record before it is processed and analyzed. By default it is set to 50 ms. This means that 50 ms worth of data samples are recorded and then handed over to the Analyzer class that starts the signal analysis in its own thread immediately. This constant can be changed by the developer if needed.

The Analyzer class is a class intended for a recorded signal with MAX_REC_TIME analysis. An instance of this class is running in its own thread, but this thread is started immediately after the recording has started and is not waiting to be created by a Recorder class instance after the MAX_REC_TIME interval. This allows us to get rid of any unwanted delays. The analyzer thread (Figure 7) is in a waiting state until it is unblocked by the Recorder thread after samples of data are recorded and buffered. After the Analyzer thread is done, it is automatically reset to a wait state and waits for another Recorder thread unblock. This behavior is provided by the class of EventWaitHandle type, which is initiated with an AutoResetevent type class, which is of course a subclass of the EventWaitHandle class. All of this is possible thanks to C# and object oriented polymorphism.

The variable that represents an instance of this EventWaitHandle class is available both to the Analyzer and Recorder class. The Analyzer thread is waiting after WainOne() method has been called for as long as it takes to Recorder class to buffer recorded data and call Set() method on this shared EventWaitHandle type object, that releases the lock in Analyzer thread required by WaitOne method. After the analysis is completed, the lock is in place right on, thanks to AutoResetEvent type of shared object instance.

Data are recorded after MAX_REC_TIME to the same source that is being read off. To avoid read/write conflicts, simple C# thread variable locks are used. All considered, even though the application is running in a number of threads (Figure 7), it is thread-safe. Last but not least, the Player class is a wrapper around WaveOut class that plays an alarm sound in a loop until stopped by the user. An instance of this singleton pattern designed class is running in its own thread as well.

### 4.3. Other Resolved Issues

**Figure 8.**
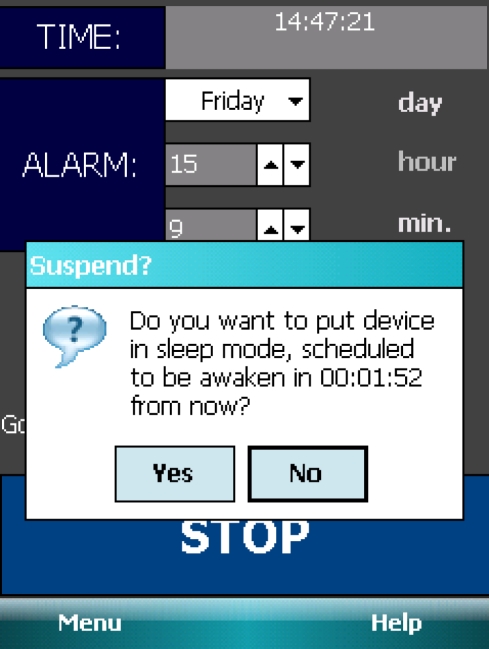
wakeNsmile application screenshot.

This section describes other issues that had to be resolved during the development of the application in order to make the application function properly. The first issue was the power management, and more specifically, the issue with device hibernation (Figure 8) and auto awakening in the 30 min time span before the set wakeup time. Neither the compact framework nor the OpenNETCF library provide a method to implement and resolve this issue. The native win32 API methods imported from the coredll.dll library had to be used. More specifically, the “CeRunAppAtTime” method that accepts as parameters win32 named events to be signaled at a specified time (Program Code 1). Using this method and time to wake the device up at a specified time as a second “CeRunAppAtTime” parameter, the device is awakened 30 min before the preset wakeup time and starts to record and analyze.

**Program Code 1.** Program code in C# for hibernation procedure of Windows Mobile device.

DateTime timeToWake = DateTime.Now;

timeToWake = timeToWake.Add(timeSpan);

long timeToWakeAsFileTimeUTC = timeToWake.ToFileTime();

long timeToWakeAsFileTimeLocal = 0;

Win32.FileTimeToLocalFileTime(ref timeToWakeAsFileTimeUTC, ref timeToWakeAsFileTimeLocal);

SystemTime timeToWakeAsSystemTime = new SystemTime();

Win32.FileTimeToSystemTime(ref timeToWakeAsFileTimeLocal, timeToWakeAsSystemTime); //Create Win32 named event hanle

nativeWaitHandle = Win32.CreateEvent(0, 0, 0, _eventName); //Start the background thread

_backgroundThread = new Thread(BackgroundThreadFunction);

_backgroundThread.Start();

Win32.CeRunAppAtTime(_wakeupEventName, timeToWakeAsSystemTime); //Run an event

## 5. Results and Discussion

This section displays and describes the results on tested subjects divided into two main—subjects with pathological sleep (group P) and subjects with normal healthy sleep (group H). The wakeNsmile application is not recommended for subjects with pathological sleep issues. For subjects with no sleep sound manifestation, the wakeNsmile application works as a normal alarm clock and wakes the user up at a specified time.

The subjects were selected to show a variety of situations for the application detection algorithm and to prove its reactions. Pathological sound manifestations in the following examples are filtered out by the pathologicals filter described in Section 3.1. The total number of four subjects out of 10 tested are described in detail in Sections 5.2 and 5.3. Table 5 show the statistical results for all tested subjects.

The wakeNsmile application was tested for functionality and for sleep stage detection accuracy. The testing methodology was created in a way to achieve the most accurate and reliable results possible. Before the test, each person was requested to fill out a questionnaire. The questionnaire asked several simple questions to detect the group to which a tested person belonged to. This was a very important phase of testing methodology because it helped us to exclude persons with pathological hypnograms.

After the first questionnaire phase of the testing methodology, real testing was only started with a collection of subjects with healthy hypnograms. In this collection, a sufficient number of tests (14 successfully finished tests) were executed to get a valid collection of results. In a second phase of testing, subjects with pathological sleep (Nos. 3, 5 and 10 in Table 5) were included in the test of the pathologicals filter efficiency and to show that the application is not recommended for users with pathological sleep (subject to specific exceptions).

For the purposes of testing, subjects were split into two main groups and subgroups, which, from the sleep sound manifestation point of view, is sufficient to classify and divide tested subjects:

- H—healthy sleep
- P—pathological sleep (S—snoring, N—no manifestations, O—other issues)

### 5.1. Problems Faced during the Testing Period

The only problem which was detected during our initial test period was the disturbance during the tests which sometimes resulted in an inaccurate sleep stage detection. The problem is caused by the way the signal is measured by the microphone. To solve this problem, we selected only those test persons who sleep alone in a room. This is a significant limitation, but work is underway to overcome this issue.

### 5.2. Healthy Sleeping Subjects

#### 5.2.1. Subject No.1 (Figure 9, male, age: 24, normal sleeping, wakeup time: 7:00, group: H)

Application log output:

2010-08-13 06:30:01,000[INFO]-Device awaken from standby mode

2010-08-13 06:34:47,000 [INFO]-Movement sound detection

2010-08-13 06:34:54,000 [INFO]-Movement sound detection

2010-08-13 06:43:20,000 [INFO]-Movement sound detection

2010-08-13 06:43:21,000 [INFO]-Starting alarm

Result: The subject was correctly woken up by the application 16 min, 39 s before the set up time. Comment: The application correctly evaluated situation and woke the user up.

#### 5.2.2. Subject No. 2 (Figure 10, female, age: 23, normal sleeping, wakeup time 6:00, group: H)

Application log output:

2010-08-10 05:30:01,000[INFO]-Device awaken from standby mode

2010-08-10 05:34:02,000 [INFO]-Movement sound detection

2010-08-10 05:35:22,000 [INFO]-Movement sound detection

2010-08-10 05:35:32,000 [INFO]-Movement sound detection

2010-08-10 05:35:33,000 [INFO]-Starting alarm

Result: The subject was correctly woken up by the application 25 min, 27 s before the set up time. Comment: The application correctly evaluated the situation and woke the user up.

### 5.3. Subjects with Pathological Sleeping Manifestations

#### 5.3.1. Subject No. 3 (Figure 11, male, age: 28, sleep with snoring, wakeup time 7:00, group: P–S)

Application log output:

2010-08-10 06:30:01,000[INFO]-Device awaken from standby mode

2010-08-10 06:35:02,000 [INFO]- Snoring detected and filtered

2010-08-10 06:35:05,000 [INFO]- Snoring detected and filtered

2010-08-10 06:35:08,000 [INFO]- Snoring detected and filtered

…

2010-08-10 06:41:58,000 [INFO]- Snoring detected and filtered

2010-08-10 07:00:00,000 [INFO]-Starting alarm

Result: The subject was woken up at the set up time 7:00, the snoring sound was filtered correctly and the user was not woken at the NREM stage, which would be unpleasant.

Comment: The application correctly evaluated the situation and did not wake the user up while snoring.

#### 5.3.2. Subject No. 4 (Figure 12, female, age: 50, no sleep sound manifestation, wakeup time 5:30, group: P–N)

Application log output:

2010-08-10 05:00:01,000[INFO]-Device awaken from standby mode

2010-08-10 05:30:00,000 [INFO]-Starting alarm

Result: The subject was woken at the set up time 5:30 because no event was recorded and detected at all.

Comment: The application correctly evaluated the situation and woke user up at the set up time 5:30. Table 4 summarizes the results of the four selected subjects.

### 5.4. Statistical Results

This chapter shows statistical results of all tested subjects including the total statistical results, healthy and pathological group’s results.

Four subjects were woken up correctly, even though two of them (Sections 5.2 and 5.3) had pathological sleep issues. These two subjects were woken up at the set up time and not sooner because the detection algorithm detected snoring sounds and classified them as not suitable for further analysis for the first subject (Section 5.3.1) and did not detect any sound for the second subject with pathological sleep (Section 5.3.2).

The pathological sleep with no sound manifestation had no movement or significant and loud breathing during the night. The application is not recommended for subjects with a pathological sleep.

The algorithm has been subjected to 19 tests on a total of 10 subjects. In 72.5% of cases, it successfully detected mild sleep stages as can be seen in the row with the “Total” summary at Table 5.

The “Total P” row provides the results summary for pathological persons as categorized by the questionnaire. While we do not recommend using the developed application for pathological persons, mild sleep stages were detected in 33% of cases in this category.

The last described row “Total H” provides a summary for the healthy persons category, which successfully detected mild sleep stages in 89% of cases. Such very good results exceeded our expectations. With such a promising quality of the developed solution we plan to start an additional predicative test where statistically strong results are anticipated.

## 6. Proactive User Adaptivity

The wakeNsmile application is developed to address on the needs of users to enjoy a happy awakening at a predefined time. The time defined by the alarm is however the latest possible time to wake the user up. We are trying to detect a body state in which the user is most likely to wake up with a smile. The time period for the detection analysis of state phases is 30 min. The application is an example of a user adaptive solution for mobile devices [16,17]. Currently, a single application is developed, but a distributed architecture version with a neural network analysis and a people database is planned for future steps to be a completely embedded solution in a Mobile UAS (User Adaptive System) Framework which grows from several previous project conjunctions [18–21]. The tests performed during this study provide very promising results, with more than a 70% accuracy in happy wake ups of test persons in the morning.

The developed application acts as a proactive solution in that it helps users enjoy a happy wake up in the most appropriate body state. User adaptivity can be however sustained by implementing a user’s stored inputs collection (user manual sets along with successfully detected mild sleep stages) to achieve a higher level of user adaptivity. Similar adaptation is used by the next intelligent alarm project called “Gently Alarm” [22].

In a wider context, a limited type of green pervasive computing system can grow from the developed application as a base for such system inputs in the form of users’ knowledge or users’ mental state during a day after a successful or unsuccessful happy wake up [23,24].

## 7. Project Extension and New Capabilities

**Figure 13.**
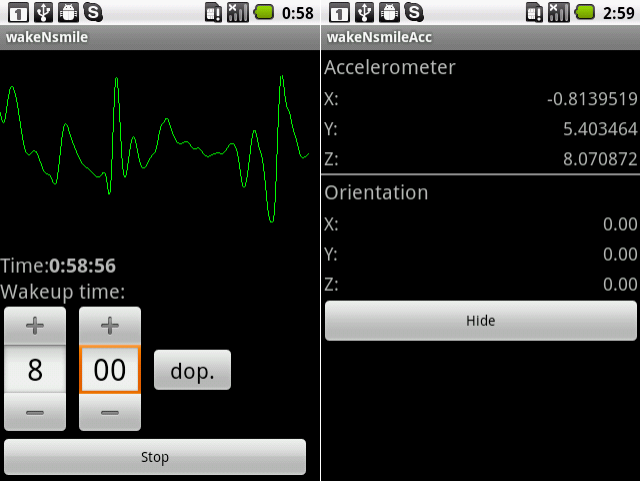
wakeNsmile and wakeNsmileAcc application on the Android platform.

The wakeNsmile project and application was developed on Windows Mobile 6.5 as an application for REM/NREM sleep stage detection. Both the platform and detection methods are being continuously developed to achieve a higher level of sleep state detection quality. Because the Windows Mobile platform has been phased out in most applications, replaced by Windows Phone 7 a new version of an application is being developed on the Android platform (Figure 13) and REM/NREM stages are detected not only by sound movement detection but by movement detection itself with the application of the devices’ built-in accelerometer sensors. This application extension provides better results than only sound input recording and detection. The application area is also being extended to epilepsy seizure detection in cooperation with a university hospital’s small children neurological department.

## 8. Conclusions

The application was tested on several subjects with correct results. First, a collection of four selected persons representing two major categories (healthy and pathological sleep) were tested and interviewed to provide information about different monitoring process of the application in real life. The application is suitable for persons with healthy hypnograms where happy wake ups were achieved in 89% of cases.

The algorithm shows one of the possible ways to achieve the aims of this project and application but to improve this algorithm further neural networks should be considered and used in this application. Neural networks fed with data from different biological events and recorded in sound format can detect an anomaly much more precisely and effectively and make a proper decision.

## Figures and Tables

**Figure 9.**
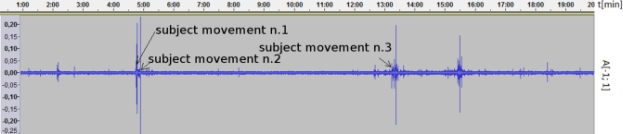
Recorded sound data of subject No. 1.

**Figure 10.**
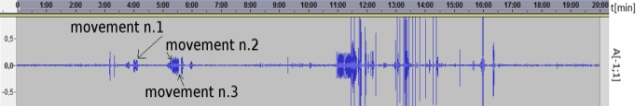
Recorded sound data of subject No. 2.

**Figure 11.**
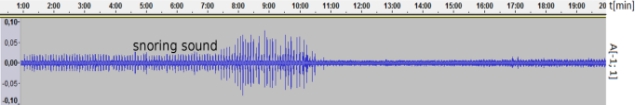
Recorded sound data of subject No. 3.

**Figure 12.**
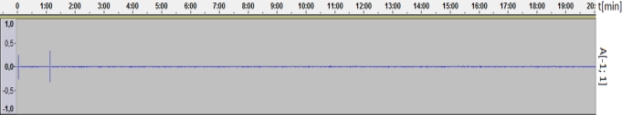
Recorded sound data of subject No. 4.

**Table 1.** Biological manifestations in sleep stages.

**Physiological Process**	**During NREM**	**During REM**
brain activity	decreases from wakefulness	increases in motor and sensory areas, while other areas are similar to NREM
heart rate	slows from wakefulness	increases and varies compared with NREM
blood pressure	decreases from wakefulness	increases (up to 30 percent) and varies from NREM
blood flow to brain	does not change from wakefulness in most regions	increases by 50 to 200 percent from NREM, depending on brain region
respiration	decreases from wakefulness	increases and varies from NREM, but may show brief stoppages (apnea); coughing suppressed
airway resistance	increases from wakefulness	increases and varies from wakefulness
body temperature	is regulated at lower set point than wakefulness; shivering initiated at lower temperature than during wakefulness	is not regulated; no shivering or sweating; temperature drifts toward that of the local environment
muscle tension	decreasing with increase of NREM	increases from NREM

**Table 2.** Snoring time differences.

**Snoring sample number (Patient num.snore number)**	**Time difference [s]**	**Percentage deviation [%]**

P1.1–2, P1.2–3, P1.3–4, P1.4–5, P1.5–6, *P1.6–7*, P1.7–8	3.2, 3.1, 3.6, 3.5, 3.5, *0.7*, 3.6	0%, 3.1%, 12.5%, 9.4%, 9.4%, 78.1%, 12.5%
P2.1–2, P2.2–3, P2.3–4, P2.4–5, P2.5–6,	2.5, 2.7, 2.8, 2.4, 2.5	0%, 8%, 12%, 4%, 0%
P3.1–2, P3.2–3, P3.3–4, P3.4–5, P3.5–6, P3.6–7	4, 3.8, 3.7, 3.9, 4, 3.9	0%, 5%, 7.5%, 2.5%, 0%, 2.5%
P4.1–2, P4.2–3, P4.3–4, P4.4–5, P4.5–6, P4.6–7	2.7, 2.8, 2.5, 2.7, 2.7, 2.6	0%, 3.7%, 7.4, 0%, 0%, 3.7%

**Table 3.** Summary of tested device parameters.

	**HTC device**				
	**Athena**	**Roadster**	**Touch HD**	**Touch HD2**	**Blue Angel**
OS WM	6.5 Prof.	6.0	6.0 Prof.	6.5 Prof.	5.0
CPU [Mhz]	624	624	528	1024	400
RAM [MB]	128	64	288	448	128
ROM [MB]	256	128	512	512	64
LCD	480 × 640	480 × 640	480 × 800	480 × 800	240 × 320

**Table 4.** Tested subject result table.

**No.**	**Gender**	**Age**	**Sleep**	**Set wakeup time**	**Wakeup time**

1	male	24	normal	7:00	6:43
2	female	23	normal	6:00	5:36
3	male	28	snoring	7:00	7:00
4	female	50	no movement	5:30	5:30

**Table 5.** Algorithm detection statistical results.

**Subject No.**	**Num. of tests**	**Group**	**Success**	**Failed**

1	4	H	100%	0%
2	2	H	100%	0%
3	2	P–S	50%	50%
4	3	H	75%	25%
5	1	P–N	0%	100%
6	1	H	100%	0%
7	1	H	100%	0%
8	1	H	100%	0%
9	2	H	50%	50%
10	2	P–S	50%	50%

Total	19		72.5%	27.5%

Total H	14	H	89%	11%
Total P	5	P	33%	67%
